# Provision of relapse prevention interventions in UK NHS Stop Smoking Services: a survey

**DOI:** 10.1186/1472-6963-10-214

**Published:** 2010-07-20

**Authors:** Shade A Agboola, Tim J Coleman, Jo A Leonardi-Bee, Andy McEwen, Ann D McNeill

**Affiliations:** 1 UK Centre for Tobacco Control Studies, Division of Epidemiology and Public Health, University of Nottingham, Nottingham, UK; 2 UK Centre for Tobacco Control Studies, Division of Primary Care, University of Nottingham, Nottingham, UK; 3 Cancer Research UK Health Behaviour Research Centre, University College London, London, UK

## Abstract

**Background:**

UK NHS Stop Smoking Services provide cost effective smoking cessation interventions but, as yet, there has been no assessment of their provision of relapse prevention interventions.

**Methods:**

Electronic questionnaire survey of 185 UK Stop Smoking Services Managers.

**Results:**

Ninety six Stop Smoking Service managers returned completed questionnaires (52% response rate). Of these, 58.3% (n = 56) ran NHS Stop Smoking Services which provided relapse prevention interventions for clients with the most commonly provided interventions being behavioural support: telephone (77%), group (73%), and individual (54%). Just under half (48%, n = 27) offered nicotine replacement therapy (NRT), 21.4% (n = 12) bupropion; 19.6% (n = 11) varenicline. Over 80% of those providing relapse prevention interventions do so for over six months. Nearly two thirds of all respondents thought it was likely that they would either continue to provide or commence provision of relapse prevention interventions in their services. Of the remaining respondents, 66.7% (n = 22) believed that the government focus on four-week quit rates, and 42.9% (14 services) believed that inadequate funding for provision of relapse prevention interventions, were major barriers to introducing these interventions into routine care.

**Conclusions:**

Just over half of UK managers of NHS Stop Smoking Services who responded to the questionnaire reported that, in their services, relapse prevention interventions were currently provided for clients, despite, at that time, there being a weak evidence base for their effectiveness. The most commonly provided relapse prevention interventions were those for which there was least evidence. If these interventions are found to be effective, barriers would need to be removed before they would become part of routine care.

## Background

The effectiveness and cost-effectiveness of UK NHS Stop Smoking Services in providing support for smokers wishing to stop have been demonstrated, with more than half of English services' clients achieving self-reported abstinence from smoking for at least four weeks[1,2] However, quitters' rates of relapse to smoking are high, with around 75% of those abstinent at four weeks after their quit date re-starting smoking by one year[1]. The use of effective treatments to reduce rates of relapse to smoking (which we refer to here as relapse prevention interventions, could greatly improve long term cessation rates for the NHS Stop Smoking Services.

A Cochrane review found no clear evidence for behavioural relapse prevention interventions being effective and only relatively weak evidence that some medications, effective for smoking cessation, may also be effective as relapse prevention interventions when provided for extended periods to smokers who had achieved short-term abstinence[3]. We recently completed a review of the evidence base for relapse prevention interventions using a similar search strategy to the Cochrane review, but restricting included studies to those involving abstinent smokers only, and synthesising data from similar follow up time points only, rather than aggregating final follow up data collected at different times as the Cochrane review did[4]. We found that, amongst individuals who had stopped smoking without any cessation support, behavioural self help relapse prevention interventions were effective and varenicline, bupropion and nicotine replacement pharmacotherapy interventions also were effective for preventing relapse to smoking amongst smokers who had achieved abstinence using drug treatments.

As little was known about either the current provision of relapse prevention interventions in UK NHS Stop Smoking Services or the feasibility of adding these to existing treatment options, we carried out a small qualitative study with a convenience sample of 16 Stop Smoking Service managers[5] to explore these factors. We found that managers had no shared understanding of either the concept of relapse prevention or the kinds of interventions that should be used to prevent relapse, but there was interest in relapse prevention interventions and a willingness to make these available to stop smoking services' clients and this was already happening in some areas. Consequently, in this study, we aimed to quantify the current provision of relapse prevention interventions within the Stop Smoking Services in the UK and ascertain the feasibility of introducing them to services where they were not currently provided. To overcome the lack of a shared understanding of relapse amongst NHS Stop Smoking Service staff, we defined relapse prevention interventions as behavioural or drug therapies delivered after acute smoking cessation treatment has ended and resulted in abstinence from smoking.

## Methods

For the benefit of international readers, Additional file 1: Box 1, gives brief details regarding the characteristics of UK NHS Stop Smoking Services [6] and all service managers were asked to complete an electronic questionnaire. From the emergent findings of our systematic review[4] and qualitative research referred to above[4,5] issues of potential importance to the provision of relapse prevention interventions were identified and, from these, a structured questionnaire (Additional File 2), was developed (which was available online at http://www.smokingcessationmanagers.org). The questionnaire was designed to obtain information pertaining to current provision of treatment for smoking cessation as well as current and future relapse prevention treatment provision. The questionnaire also asked about the feasibility of providing the most promising relapse prevention interventions (identified from the reviews and qualitative research as NRT, bupropion, varenicline, group and individual behavioural counselling, and NRT combinations) in routine clinical practice. A clear definition of relapse prevention was given, namely "***Relapse Prevention Interventions (or Relapse Prevention Treatments) are behavioural or drug therapies delivered after acute smoking cessation treatment has ended and resulted in abstinence from smoking. Relapse Prevention Interventions therefore seek to reduce relapse to smoking among abstinent smokers"***. We also distinguished relapse prevention interventions from interventions delivered to smokers who had briefly lapsed to smoking (i.e. were not abstinent) and which aimed to prevent a full relapse to smoking; respondents were asked to indicate provision of any such lapse-orientated interventions/treatments. An email with a flyer advertising the survey and a link to the survey homepage was sent out to a total of 185 managers in December 2008. Non-respondents were followed up via a reminder email and telephone call inviting them to visit the survey homepage and complete the survey. Responses were anonymous and data were summarised descriptively using SPSS version 16.0 for Windows[7]. No hypotheses testing statistical analyses were performed, but some comparisons are presented for descriptive purposes.

## Results

A total of 96 managers completed the survey (52% response rate). Fifty four managers responded to the first survey email and completed the online questionnaire. A further 42 respondents completed the survey following the reminder email and telephone call.

### Current provision of smoking cessation and relapse prevention treatments

The vast majority of services provided both behavioural and pharmacotherapy interventions for initial cessation treatment. More than half of respondents, 58.3% (n = 56) reported that their services currently provided relapse prevention interventions with behavioural interventions being preferred over the pharmacotherapies (Table 1). Of those providing relapse prevention interventions, 60.7% (n = 34) stated that these were offered to abstinent smokers for as long as these clients perceived they required them, 25.0% (n = 14) for 3-6 months and one service (1.8%) did so for three months or less. Of the 40 services reporting that they did not currently provide relapse prevention interventions, 42.5% (n = 17) had provided such interventions in the past and cited the following reasons for no longer offering this kind of support: pressure to meet government targets, poor client attendance, inadequate funding, and a belief that relapse prevention interventions are ineffective (Table 2).

**Table 1:** Provision of acute cessation treatment and relapse prevention treatment in UK NHS Stop Smoking Services

	Type of Treatment % (n)					
	**Individual behavioural counselling**	**Group behavioural counselling**	**Telephone counselling**	**Nicotine Replacement Therapy**	**Bupropion**	**Varenicline**

**Acute Cessation**	99% (95)	87.5% (84)	81.2% (78)	97.9% (94)	86.5% (83)	92.7% (89)

**Relapse Prevention**	73.2%(41)	53.6% (30)	76.8% (43)	48.2% (27)	21.4% (12)	19.6% (11)

**Table 2:** Reasons for discontinuation of relapse prevention treatment

Reason	% (n)
Pressure to meet targets	64.7% (11)

Poor client attendance	70.6% (12)

Inadequate funding	29.4% (5)

Ineffectiveness of relapse prevention interventions	17.6% (3)

Managers that responded to the first survey email were compared to those that completed the survey after a reminder telephone call. There was no association between timing of responses and relapse prevention provision (Odds ratios (OR) 0.77; 95% confidence interval (CI) 0.34 to 1.75).

### Current provision of treatment for brief lapses

A large percentage of UK NHS Stop Smoking Services, 77.1% (n = 74) also provided treatment for clients who had suffered a brief lapse to smoking. Of these, 72.9% of services (n = 70) indicated the types of support provided: one to one sessions (32.8%, n = 23); rolling groups and drop-in sessions (7.1%, n = 5); telephone support (8.5%; n = 6); combinations of pharmacotherapy, telephone support, and one to one behavioural counselling (15.7%, n = 11); while 35.7% (n = 25) services continued acute cessation treatment. Although managers who provided relapse prevention interventions were also more likely to report providing treatment for brief lapses than those not providing relapse prevention interventions, this was not statistically significant (OR) 1.55; 95% CI 0. 59 to 4.04, P = 0.37).

Managers who responded to the first survey email did not differ from those that responded to the telephone reminder in terms of treatment of brief lapses (OR 1.92; 95% CI 0.70 to 5.26).

### Feasibility of providing relapse prevention interventions

Respondents were asked to indicate the likelihood of future relapse prevention intervention provision within their service. Managers who had indicated that relapse prevention interventions were not currently provided were asked to indicate the likelihood of providing these in the future and those already providing relapse prevention interventions were asked about the likelihood of continuing this. Nearly two - thirds, 65.6% (n = 63) of managers thought it very likely or likely that they would continue to provide, or start to provide relapse prevention interventions in their services, while 34.4% (n = 33) of services were not sure or thought it unlikely that they would provide these interventions in the future. There was no association between managers who responded to the first survey email and those who responded to the second survey email in terms of likelihood of future relapse prevention provision (OR 1.05; 95% CI 0.45 to 2.46).

Managers who wished to discontinue or not to begin relapse prevention support cited the following reasons: cessation orientated targets focussed on four week quit rates (66.7%, n = 22); inadequate funding (42.9%, n = 14); and the fact that clients had usually relapsed before they re-contacted the cessation service (24%, n = 8).

These 33 respondents who indicated that they were not sure or thought it unlikely that their services would provide relapse prevention interventions in the future, were then asked to assume that barriers to provision of these interventions were removed, and to hypothesise, in this instance, which of these interventions they might encourage their NHS Stop Smoking Service to offer to abstinent quitters after smoking cessation treatment. Table 3 reports their responses.

**Table 3:** Likelihood of providing relapse prevention interventions in the absence of barriers

Intervention	Very Likely/Likely % (n)	Not sure/Unlikely/Definitely not % (n)
**Individual Counselling**	78.8% (26)	18.2% (6)

**Group counselling**	72.7% (24)	27.2% (9)

**NRT**	57.6% (19)	33.3% (11)

**Bupropion**	21.2% (7)	72.7% (24)

**Varenicline**	24.2% (8)	75.8% (25)

**NRT combinations**	54.5% (18)	39.4% (13)

## Discussion

### Summary of findings

About half of the services surveyed reported providing relapse prevention interventions despite, at the time of the survey, their being only a weak evidence base for their effectiveness and no guidance as to whether or not this kind of support should be provided within the UK Stop Smoking Services. The relapse prevention interventions provided most frequently were telephone and individual behavioural counselling although, at the time of the survey there was no evidence for the effectiveness of these interventions (i.e. from the Cochrane review) and this has not changed with our subsequent review. Many managers were positive about either offering relapse prevention interventions within the NHS or potentially introducing these in the future and the most commonly cited reasons for not providing or wanting to provide these interventions were that: NHS Stop Smoking Services' targets are focussed on achieving short periods of cessation by smokers and inadequate funding for relapse prevention support, rather than the relatively weak current[3,8] evidence base for the effectiveness of relapse prevention interventions.

### Strengths and Limitations

Approximately 50% of survey recipients did not respond and, consequently, the reported rates of interest in and provision of relapse prevention interventions may be overestimated as managers with little interest in providing relapse prevention interventions might have been less likely to respond. A greater response rate may have produced more varied results; non-responders to the survey may have different experiences of providing relapse prevention interventions in their services, although a comparison of the two waves of responses to the survey showed that there were no differences in variables relevant to the survey. Also amongst respondents, some managers may have been unwilling to reveal that they do not provide some form of relapse prevention intervention in their services. However, we believe that biases in the reporting amongst respondents are unlikely as, at the time of the survey, using such interventions was not standard stop smoking service practice and no official guidance recommended their use. The questionnaire used mainly closed ended questions as these are less time consuming to complete and help to optimise completion rates[9] but provided an "other" option for respondents to provide additional information where none of the available options fit the manager's response. Closed ended questions may restrict the respondents to the choices provided, but we do not believe this is the case, because we provided respondents with an "other" option with space for free text which gave managers the opportunity to expand on their response, and greater freedom of expression[10].

Despite these limitations, we believe that the survey is important; it is the first study to investigate the provision of relapse prevention interventions in UK NHS Stop Smoking Services and now that there is evidence that some of these interventions may be effective[4], findings can be used to facilitate the introduction of evidence based ones into the services.

There is no previous research on this topic from the UK and, we believe, internationally as the World Health Organization report on the global tobacco epidemic provides no information on the provision of relapse prevention interventions [11]. We could identify no similar studies internationally which described the provision of relapse prevention interventions in routine clinical practice, so it is difficult to make comparisons. The reasons why such a high proportion of managers reported offering relapse prevention interventions which had no proven evidence base are unknown and may be worth further exploration. For example, this could reflect a desire to do 'something' to remedy high relapse rates from acute cessation treatment, or their anecdotal experience of the effectiveness of relapse prevention interventions, or a perception that smokers appreciate being offered such treatment. As managers favoured behavioural over drug treatments, this suggests that, with smoking cessation services configured as they are currently, it may be difficult to encourage the use of pharmacological interventions for relapse without there being clear guidance on this. Almost 77% of respondents working in services that delivered relapse prevention interventions provided telephone counselling which could be explained by the finding that most services provided telephone support to aid cessation and this may have been relatively easy and inexpensive to extend. Clearly, integrating effective pharmacological interventions into routine clinical practice has cost implications and might be more challenging.

## Conclusions

Current provision of acute cessation treatment largely reflected UK guidance (NICE, SCOTTISH NICE[12], DH Monitoring guidance[13]) and our survey suggests that similar evidence based guidance is needed to support managers wishing to provide relapse prevention interventions in order to ensure that, in the future, the most promising and effective interventions can be introduced into routine care. Further research is required to solidify current evidence on the effectiveness of relapse prevention interventions and then to explore further the feasibility and cost - effectiveness of introducing these in the NHS Stop Smoking Services. Should relapse prevention interventions be found to be cost - effective and acceptable for use by abstinent smokers in the NHS Stop Smoking Services the perceived barriers would need to be removed.

## Competing interests

Within the last five years Tim Coleman has undertaken consultancy work for Pierre Fabre Laboratories, France and also Johnson & Johnson. Both companies produce nicotine replacement therapy. Andy McEwen has received travel funding, honorariums and consultancy payments from manufacturers of smoking cessation products (Pfizer Ltd, Novartis and GSK Consumer Healthcare Ltd). He also receives payment for providing training to smoking cessation specialists and receives royalties from books on smoking cessation.

The other authors declare that they have no competing interests.

## Authors' contributions

All authors contributed to design of the survey questionnaire. SA carried out the survey analysis and wrote the first draft of the paper. All authors read and approved the final manuscript.

## Pre-publication history

The pre-publication history for this paper can be accessed here:

http://www.biomedcentral.com/1472-6963/10/214/prepub

## Supplementary Material

Additional file 1 **Box 1 - Characteristics of NHS Stop Smoking Services**. The box provides a background, details of service provision and other characteristics of NHS Stop Smoking Services. Click here for file

Additional file 2 **Survey Questionnaire**. This file contains the entire survey questionnaire. Click here for file
