# Supplementary material for: Provision of relapse prevention interventions in UK NHS Stop Smoking Services: a survey
Source: BMC Health Serv Res. 2010 Jul 20;10:214. doi: 10.1186/1472-6963-10-214 (PMC2912902; doi:10.1186/1472-6963-10-214)
Supplement: Additional file 1 — Box 1 - Characteristics of NHS Stop Smoking Services. The box provides a background, details of service provision and other characteristics of NHS Stop Smoking Services. [file 1472-6963-10-214-S1.DOC]

**Box 1**

Characteristics of NHS Stop Smoking Services

| **Background**   - Established in 1999 in the most disadvantaged areas in England - Rolled out across the UK from 2000 - Represent a unique national initiative to provide support for smokers motivated to quit[1]   **Service provision**   - Service provision framework is based on an evidenced based approach to treating dependent smokers[2] - Usually involves regular meetings (one to one or in groups) with a trained adviser, using structured withdrawal-oriented behavioural support combined with smoking cessation medications[1] - Smoking cessation medications are usually offered on the basis of an abstinent-contingent treatment programme involving an initial course of two to four weeks, followed by further prescriptions if the quit attempt is continuing[3]. - If a smokers’ attempt to quit is unsuccessful, advisers can use discretion and professional judgement when considering whether a client is immediately ready to receive support to attempt to stop smoking again[4]   **Training**   - Smoking cessation staff come from a wide variety of backgrounds - They are trained in the provision of treatments to help with stopping smoking - This training is currently being standardised in England through a newly established National Centre for Smoking Cessation and Training   **Targets**   - For the first ten years, targets were set within England for the numbers of smokers attending the services who set a quit date and who quit smoking four weeks after the start of treatment - Smoking cessation services are currently expected in the course of a year to treat 5% of their local population , in line with best practice recommendations contained within National Institute for Health and Clinical Excellence programme guidance for smoking cessation[3] |
| --- |

Reference List

1. Bauld L, Bell K, McCullough L, Richardson L, Greaves L. The effectiveness of NHS smoking cessation services: A systematic review. Journal of Public Health 32[1], 71-82. 2009.
Ref Type: Journal (Full)

2. West R, McNeill A., Raw M.. Smoking cessation guidelines for health professionals: An update. Thorax 55, 987-99. 2000.
Ref Type: Journal (Full)

3. NICE public health guidance 10: Smoking cessation services in primary care, pharmacies, local authorities and workplaces, particularly for manual working groups, pregnant women and hard to reach communities. 2-87. 2008. 7-6-2010.
Ref Type: Report

4. NHS Smokefree. NHS Stop Smoking Services Service and Monitoring Guidance. 1-106. 2009. Department of Health. 17-6-2010.
Ref Type: Report
