# Supplementary material for: Provision of relapse prevention interventions in UK NHS Stop Smoking Services: a survey
Source: BMC Health Serv Res. 2010 Jul 20;10:214. doi: 10.1186/1472-6963-10-214 (PMC2912902; doi:10.1186/1472-6963-10-214)
Supplement: Additional file 2 — Survey Questionnaire. This file contains the entire survey questionnaire. [file 1472-6963-10-214-S2.DOC]

**Relapse Prevention Intervention Questionnaire**

***This anonymous questionnaire seeks information about Relapse Prevention Interventions (which are also known as Relapse Prevention Treatments) that your smoking cessation service (SCS) currently offers or has previously offered. It should be completed by the person who manages or runs this service within your Primary Care Trust (PCT). If you are not the service manager/coordinator, then please pass this on to him or her.***

***Before we can ask questions about Relapse Prevention Interventions we need to ask you a few questions about your current service provision to put your answers in context (i.e. support you currently provide to help smokers quit).***

**Section 1: Provision of Smoking Cessation Interventions**

1. What types of behavioural smoking

| *Individual behavioural counselling*…………………………………………….. |  |
| --- | --- |
| *Group behavioural counselling*……………………….. |  |
| *Self-directed sessions using computer software*……………………………………………….. |  |
| *Telephone advice/counselling*………............................ |  |
| *Self-help materials (i. e. booklets)*…………………….. |  |
| *Peer led sessions*………………………………............. |  |
| *Other (specify below)*…………………………………... |  |

cessation interventions are delivered by

your SCS?

**(tick all that apply)**

………………………………………………………………………………………………………….

2. If your service delivers

| *Open groups (clients can join and leave at any time)*……………………………… |  |
| --- | --- |
| *Closed groups (fixed number of sessions that run sequentially; usually only joined at the first one)*………………………….. |  |
| *Other (specify below)*…………………… |  |

advice/counselling in groups

what types of group treatments does

your service provide?

***(tick all that apply)***

………………………………………………………………………………………………………….

3. If your service delivers advice/counselling

in groups, on average

how long are group sessions? … … …. .. ...………………………………………….*Minutes*

4. Is there a specific number of group sessions

| *Yes*…………………………………................... |  |
| --- | --- |
| *No*……………………………………………… |  |

that constitutes a complete course of treatment?

***If your answer above is ‘yes’, please go to question5. If ‘no’, go to question 6***

5. How many sessions constitute a

complete course of treatment? ....………………………………*Sessions*

6. If your service delivers advice/counselling

to individuals, on average

how long are individual sessions? ………………………………….Minutes

7. If your service provides individual counselling, is there a specific number of individual sessions that constitutes

| *Yes*…………………………….. |  |
| --- | --- |
| *No*……………………………... |  |

a complete course of treatment?

***If your answer above is ‘yes’, please go to question 8. If ‘no’ go to question 9***

8. How many individual sessions constitute

a complete course of treatment? .....…………................................*Sessions*

9. Roughly, what percentages of

| *Clients receiving group support*……………. *%* |
| --- |
| *Clients receiving individual support*………… *%* |

clients attending your service receive

individual or group support?

10. Which of the following drugs

| *Bupropion*……………………………………….. |  |
| --- | --- |
| *Varenicline*……………………………………… |  |
| *Nicotine replacement therapy*…………………... |  |
| *Combination NRT e.g. patch+oral product)*…………………………… |  |
| *Other (specify below)* |  |

does your service recommend to

clients?

(***tick all that apply)***

………………………………………………………………………………………….........................

11. Which of the following drugs can be

| *Bupropion*………………………………………... |  |
| --- | --- |
| *Varenicline*………………………………………. |  |
| *Nicotine replacement therapy* ………………. |  |
| *Other (specify below)*……………………………. |  |

issued directly to clients

attending your service

(e.g. by PGD, voucher

or prescription)?

***(tick all that apply)***

…………………………………………………………………………………………………………

**Section 2: Provision of Relapse Prevention Interventions**

*This section asks about relapse prevention treatments provided by your service to abstinent quitters.*

***Relapse Prevention Interventions (or Relapse Prevention Treatments) are behavioural or drug therapies delivered after acute smoking cessation treatment has ended and resulted in abstinence from smoking. Relapse Prevention Interventions therefore seek to reduce relapse to smoking among abstinent smokers. We are distinguishing relapse prevention interventions from interventions that aim to prevent a lapse becoming a full relapse to smoking (such interventions are addressed in questions 19 and 20)***

12. Does your service provide relapse

| *Yes*………………………………………………. |  |
| --- | --- |
| *No*……………………………………………….. |  |

prevention interventions to **abstinent quitters?**

***If answer above is ‘yes’ go to question 15 if ‘no’, go to question 13***

| *Yes*………………………………………………. |  |
| --- | --- |
| *No*……………………………………………….. |  |

13. Has your service ever provided relapse prevention interventions to **abstinent quitters** in the past?

***If answer above is yes, please go to question 14, if ‘no’ go to question 19***

14. Please indicate the reasons why you no longer

| *Poor client attendance*………. |  |
| --- | --- |
| *Lack of relapse prevention training courses for staff*…….. |  |
| *Inadequate funding*………….. |  |
| *Relapse prevention treatments are not effective*……………… |  |
| *Pressure to meet Department of Health Targets*…………….. |  |
| *Other (specify below)*………... |  |

provide relapse prevention interventions.

***(tick all that apply)***

………………………………………………………………………………………………………….

***Now go to question 19.***

15. What types of relapse prevention interventions do you

provide to **abstinent quitters**?

| *NRT*………………………... |  |
| --- | --- |
| *Bupropion*……………………. |  |
| *Varenicline*…………………... |  |
| *Individual behavioural counselling*…………………... |  |
| *Group behavioural counselling* |  |
| *Telephone contact*………….. |  |
| *Self-help materials*…………… |  |
| *Regular motivational letters enquiring as to progress…* |  |
| *Other (specify below)*………... |  |

***(tick all that apply)***

………………………………………………………………………………………………………….

16. How soon after completion of the acute

| *Immediately* |  |
| --- | --- |
| *After a period of time has elapsed* |  |

smoking cessation treatment can an **abstinent**

**quitter** access relapse prevention

interventions from your service?

**(tick one box)**

17*.* If you ticked “*after a period of time has elapsed*”,

please specify the length of this period  …………………………………….*Weeks*

18. For how long are relapse prevention interventions offered to **abstinent quitters** who received smoking cessation treatment from your service?

| *3 months or less*………………... |  |
| --- | --- |
| *Greater than 3 months and up to 6months*…………………………... |  |
| *Indefinitely*……………………. |  |
| *Other (specify below)*………… |  |

**(tick one box)**

………………………………………………………………………………………………………….

19. Does your service offer any

intervention for someone who

| Yes…………………………………….. |  |
| --- | --- |
| No……………………………………… |  |

has experienced a brief lapse

to smoking to prevent full blown

relapse?

20. If yes, please state what intervention is offered?

…………………………………………………………………………………………………………

**Section 3: Feasibility of relapse prevention interventions**

*This section asks about the feasibility and potential challenges of introducing / continuing to provide relapse prevention interventions within the routine care provided by your SCS.* ***Relapse prevention interventions seek to reduce relapse to smoking among abstinent smokers.***

21. If you are currently offering relapse prevention interventions, under current circumstances, how likely is it that your stop smoking service might continue to provide relapse prevention interventions to abstinent quitters?

| *Very likely*……………………… |  |
| --- | --- |
| *Likely*…………………………... |  |
| *Not sure*………………………... |  |
| *Unlikely*………………………... |  |
| *Definitely* not……………….. …. |  |

**(tick one box)**

22. If you are currently not offering relapse prevention interventions, under current circumstances, how likely is it that your

| *Very likely*……………………… |  |
| --- | --- |
| *Likely*…………………………... |  |
| *Not sure*………………………... |  |
| *Unlikely*………………………... |  |
| *Definitely* not……………….. …. |  |

stop smoking service might start to provide relapse prevention interventions

to abstinent quitters?

**(tick one box)**

***If your answer to either question 21 or 22 above is ‘not sure’, ‘unlikely’ or’ definitely not’, please go to question 23, otherwise you are now finished.***

23. Please indicate the reasons why you are not sure or do not believe it likely that relapse prevention interventions will be provided by your service in the future.

| *Inadequate funding*…………….. |  |
| --- | --- |
| *DOH focus on four-week quits, rather than long term cessation*…... |  |
| *Clients usually relapse before they contact the service*………………… |  |
| *Few clients contact the service after acute smoking cessation treatment whilst still abstinent*………………. |  |
| *Inability to provide drug treatment within the service*………………… |  |
| *Other (specify below)*…………….. |  |

***(tick all that apply)***

…………………………………………………………………………………………………………

24. Assuming that the above issues were resolved, how likely

is it that the following interventions **could** be offered

to abstinent quitters who have completed smoking cessation

treatment -as a form of relapse prevention in your service?

| ***Intervention*** | ***Very likely*** | ***Likely*** | ***Not sure*** | ***Unlikely*** | ***Definitely not*** |
| --- | --- | --- | --- | --- | --- |
| *NRT* |  |  |  |  |  |
| *Varenicline* |  |  |  |  |  |
| *Bupropion* |  |  |  |  |  |
| *Group counselling* |  |  |  |  |  |
| *Individual counselling* |  |  |  |  |  |
| *NRT combinations* |  |  |  |  |  |
| *Other relapse prevention interventions(explain below)* |  |  |  |  |  |

………………………………………………………………………………………………………….

***25. If you answered ‘probably not’ or ‘definitely not’, for any of the listed interventions please provide reasons below.***

**………………………………………………………………………………………………………….**

**………………………………………………………………………………………………………….**

**………………………………………………………………………………………………………….**

**You are now finished. Thank you very much for your help.**
